# Building a community-centered clinical research center in an underserved New York City neighborhood to enhance access to research, equity, and quality of care

**DOI:** 10.1017/cts.2025.23

**Published:** 2025-02-10

**Authors:** Amin Yakubov, James Holahan, Aaron Lord, Melanie Jay, Rachel Gross, Celia Engelson, Zariya Alvarez, Miguel Rodriguez, Leomaris Caba Caceres, Michael Reyes, Emily Drum, Xiaoting Xing, Rosario Medina, Shilpa Londhe, Brita Roy, Imad Alsayed, Gabrielle Gold-von Simson, Miriam A. Bredella

**Affiliations:** 1 NYU Langone Health – Brooklyn, Brooklyn, NY, USA; 2 NYU Clinical & Translational Science Institute, New York, NY, USA; 3 NYU Langone Health, New York, NY, USA; 4 Department of Neurology, NYU Grossman School of Medicine, New York, NY, USA; 5 Department of Medicine, NYU Grossman School of Medicine, New York, NY, USA; 6 Department of Pediatrics, NYU Grossman School of Medicine, New York, NY, USA; 7 Department of Population Health, NYU Grossman School of Medicine, New York, NY, USA; 8 Department of Radiology, NYU Grossman School of Medicine, New York, NY, USA

**Keywords:** Clinical research center, New York City, health professional shortage area, pipeline program, research priority model

## Abstract

Access to an academic clinical research center (CRC) in health professional shortage areas (HPSA) can help address healthcare disparities and increase research accessibility and enrollment. Here we describe the development of a community-centered CRC in the underserved area of Sunset Park, Brooklyn, New York, centered within a larger academic health network and the evaluation of its outcomes within the first two years. In addition to resources and space, establishment of the CRC required a culturally competent and multilingual team of healthcare professionals and researchers and buy-in from the community. Between 1/2022 and 12/2023, the CRC opened 21 new trials (10 interventional and 11 noninterventional) with greater than 500 participant visits that reflect the racial and ethnic diversity of the community. These participants represent 110 distinct zip codes; 76% of these zip codes are underserved and designated HPSA. 60% self-identified as non-White and 20% identified as Hispanic, with 12 other distinct ethnicities represented. 28% of participants speak 11 languages other than English. Community-based CRCs can be created with sustainable growth to align with the mission of the National Institutes of Health and U.S. Food and Drug Administration to meet the ever-growing clinical, social, and research needs of the communities they serve.

## Introduction

Clinical research centers (CRCs), funded in part through the National Institutes of Health’s (NIH) National Center for Advancing Translational Sciences (NCATS) and the Clinical and Translational Science Award (CTSA) program [1], play a crucial role in conducting research, particularly in underserved populations. CRCs provide facilities and expertise to support inpatient, outpatient, and community-based research [2]. To date, there are no guidelines for establishing CRCs, especially in resource-poor, health professional shortage areas (HPSAs). Here we describe the development of a community-centered CRC with the mission of providing robust infrastructure and a dedicated and experienced team of healthcare professionals and researchers.

New York University Langone Hospital–Brooklyn (NYULHB) is a full-service, 450-bed academic hospital in the Sunset Park neighborhood of Brooklyn in New York City and lies within a designated HPSA. This neighborhood lacks sufficient primary care providers and is considered a Medically Underserved Area/Population, defined by the Health Resources and Services Administration, as it includes those who experience homelessness, are Medicaid eligible, and low income [3]. The Family Health Centers (FHCs) of NYULH, one of the largest federally qualified health center networks in the country, provide high- quality health care and social support services to this community [4]. Sunset Park’s population of over 132,000 individuals are 68% non-White (15% Asian, 22% Black or African American, 29% Latino, 2% Other) and 40% foreign-born [5,6,7]. Over 50% of the households speak predominantly Chinese, Spanish, or Russian. 22% of the residents do not have health insurance and report going without medical care, exacerbating outcomes in cardiovascular disease, cancer, maternal and child health, and mental health [5,6,7]. Most individuals in its catchment area are Medicaid/Medicare beneficiaries or uninsured, representing the densest noncommercial payor community for nonpublic hospitals in the country [4]. While our hospital and the FHCs provide outstanding clinical care for this community, no dedicated research infrastructure or CRC existed to provide access to all translational phases of research.

In March 2020, in response to the COVID-19 pandemic, the U.S. Food and Drug Administration (FDA) issued an interim guidance document, “Conduct of Clinical Trials of Medical Products During the COVID-19 Public Health Emergency,” providing direction to organizations and sponsors on adopting new ways to conduct clinical trials, including enhanced utilization of technology, provision of medication and interventions by mail, and a broadening of the types of sites at which visits can occur [8]. As a result of lessons learned during the pandemic, as well as concerns among the research community regarding decreased local participation [9], the FDA issued updated draft guidance in May 2023, “Decentralized Clinical Trials for Drugs, Biological Products, and Devices [10].” It defined decentralized clinical trials (DCTs) as studies in which the need for participants to physically access hospital-based trial sites is reduced or eliminated [11]. This guidance endorses diversity and inclusiveness in trial populations by addressing social determinants of health (SDOH), including the time, cost, and logistical hurdles of traveling to a designated trial center to receive study-related treatment and interventions [12]. Following such guidance, visits can be conducted closer to home, either through a community partner or within the home itself using telehealth.

The implementation of the Brooklyn CRC included three long-term aims: 1) expand research to Brooklyn’s underserved populations; 2) foster research that is specifically relevant to Brooklyn communities and addresses their needs; and 3) train culturally competent research faculty and staff. To facilitate clinical research in Sunset Park and surrounding neighborhoods, we established a community-centered CRC with research infrastructure embedded within the larger health system research network and aligned with multisector collaborations across community, healthcare, and municipal partners.

## Methods

In 2022, a CRC was created under the leadership of the NYULH Office of Science and Research (OSR), the NYU Clinical & Translational Science Institute (CTSI), and Beyond Bridges, a landmark initiative that aims to equitably improve health and well-being among a multiethnic, multilingual, low-income immigrant community in Sunset Park. Beyond Bridges is working to develop a robust community-clinic linkage model that coordinates accessibility of health and social care to create the enabling conditions that foster community health and well-being. The initiative is composed of six integrated pillars including: 1) Community Partnerships and Care Transitions; 2) Technology; 3) Clinical; 4) Evaluation and Replicability; 5) Research; and 6) Education.

In line with this mission, we executed a strategic plan to expand research capabilities at the medical center with the goal of supporting clinical and translational research. A research assessment was conducted in the form of surveys and interviewers of investigators, community-based clinicians, staff and local organizations on the barriers to expanding research. Developing a network of CRCs ensures that we promote high quality and harmonized clinical research across our enterprise, while providing a training ground for the next generation of clinical trialists and research staff [11]. Croghan et al identified key elements in developing a CRC to advance research in academic settings. These eight elements include 1) investigator training in research; 2) administrative/coordinator support; 3) resource allocation for start-up; 4) space needs; 5) marketing plan; 6) standard charge rate; 7) building on existing infrastructure; and 8) continued education and training as well as skill specific certifications [13]. The structure developed at our CRC follows this model with two key differences. First, instead of a marketing plan, we optimize and engage community partners through the Beyond Bridges “Community Engagement Pillar.” This pillar is comprised of diverse community-engaged researchers and guided by a Community Advisory Board (CAB). The CAB meets monthly to provide input on various initiatives, including the development of the CRC and specific study related recruitment and retention strategies. This ensures that our promotion of CRC activities is bidirectional and informed by community. Second, our CRC expands on continuing education and training for research staff, including for community health workers (CHWs) involved in research [14]. This infrastructure allows us to maximize our community involvement and broaden access to novel treatments and interventions among a diverse community.

The primary mission of the CRC was to develop infrastructure and personnel required to grow clinical research in Brooklyn, while simultaneously expanding access to cutting-edge clinical trials to underserved populations. Integral to this mission was the development of a community-clinic linkage model of health care, addressing community and clinical needs through the lens of a SDOH framework. Our linkage model focuses on building integrated bi-directional relationships between our CRC and local community-based organizations to improve health outcomes. A four-pronged approach in establishing the CRC was utilized: 1) a Kaplan & Norton balanced scorecard model; 2) a research priority model; 3) a research operational model; and 4) an evaluation and continuous quality improvement plan.

### Kaplan & Norton balanced scorecard model

**Figure 1.**
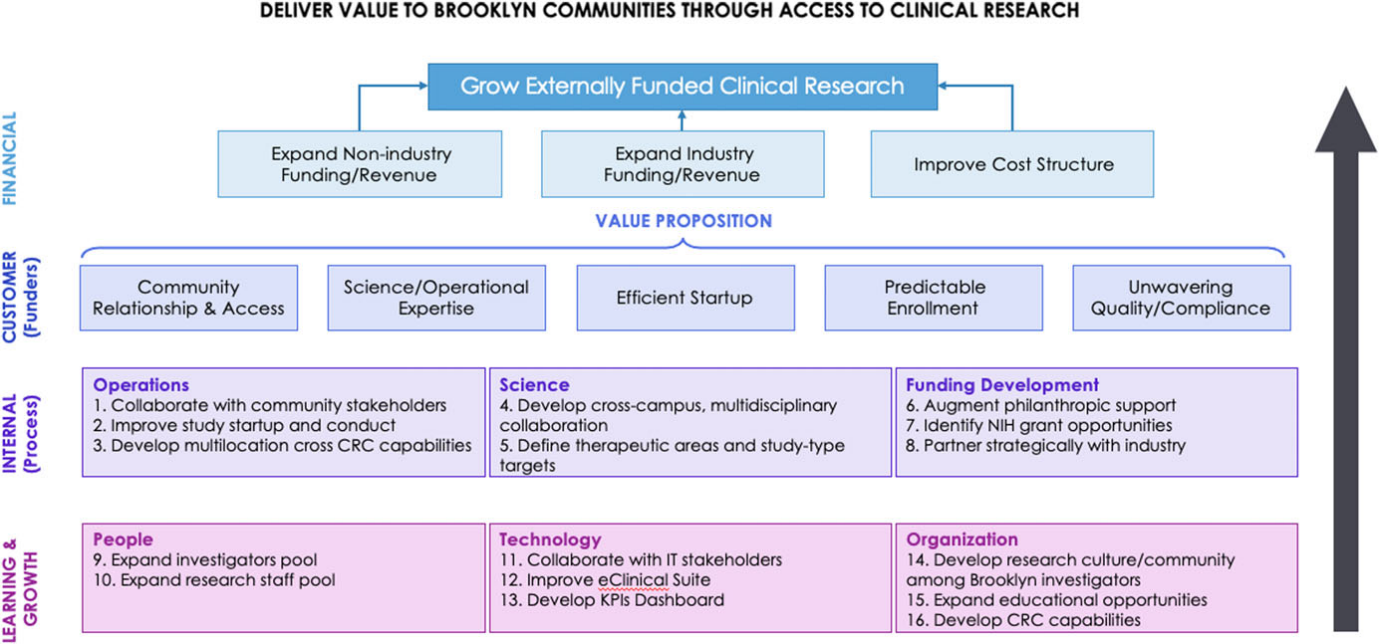
Kaplan & norton balanced scorecard (16-point strategy). Model for clinical research expansion into community, incorporates collaborating with community partners; improving study start-up; developing collaborations between pillars and departments; defining disease-focused areas; augmenting philanthropic support; partnering with industry pharmaceutical companies; expanding investigator and research professional pools; improving our technology and measurement of key performance indicators through the use of dashboards; developing and fostering a research culture in brooklyn; expanding workforce development in research; and establishing the first NYU affiliated community-centered CRC.

A Kaplan & Norton balanced scorecard model was developed along with a 16-point strategy map, building the framework for research expansion in Brooklyn (Figure 1). The scorecard is a business management framework that translates the strategic organizational mission into a specific set of performance goals and measures. Instituting the four perspectives, Financial, Customer, Internal Processes, and Organizational Capacities of Learning and Growth, an organization can align its objectives and targets, monitor performance, and drive continuous improvement over time [15,16]. We adapted this model to the research setting to meet the unique needs of clinical and translational research [17,18]. The financial perspective establishes metrics to assess financial sustainability, such as clinical trial and grant revenue. The customer perspective measures patient and provider satisfaction, quality metrics, and access to care. The internal processes perspective assesses workflows, productivity, resource utilization, and quality improvement. Lastly, the organizational capacities of learning and growth perspective improves on human capital, technology, and infrastructure with metrics that evaluate staff training, technology adoption, and innovative initiatives.

We created a 16-point strategy informed by the Kaplan & Norton balanced scorecard model to ensure expansion of community access to clinical research by: 1) partnering with community, healthcare, and municipal organizations; 2) accelerating study startup; 3) developing multilocation cross CRC research capabilities; 4) facilitating collaborations between pillars and across departments; 5) developing cross-campus multidisciplinary collaborations; 6) defining disease-focused priority research areas; 7) augmenting philanthropic support; 8) identifying NIH grant opportunities; 9) seeking support from industry pharmaceutical companies; 10) expanding the investigator research workforce; 11) creating a diverse pipeline of research professionals and expanding educational opportunities; 12) collaborating with IT stakeholders; 13) improving clinical research technologies; 14) evaluating key performance indicators; 15) establishing a community-centered CRC; and 16) fostering a research culture in Brooklyn (Figure 1). Surveys and interviews were conducted with stakeholders across the NYULHB research community to further inform and augment this strategy.

### Research priority model

We developed a research priority model to prioritize studies in disease areas impacting the Sunset Park community, informed by poor outcomes in these areas as described in the community health profile of Sunset Park: Brain and Mental Health; Maternal and Early Childhood Health; Cancer Treatment and Prevention; and Cardiometabolic, Pulmonary, and Infectious Disease [5]. To date, 90% of studies conducted at the CRC reflect our research priority areas. Research priorities are reviewed yearly to ensure new disease areas are added as disease prevalence rates change. Furthermore, we engage with our community partners to identify emerging health concerns and SDOHs, incorporating community and patient feedback into our priorities. This collaborative approach ensures that our research remains relevant and impactful in addressing the pressing issues facing the community, including the unique immigrant context that contributes to the drivers of incidence and treatment of health conditions (e.g., social isolation, poverty, cultural stigma, and trauma associated with immigration).

### Research operational model

The research operational model established the operational priorities and expectations required to conduct clinical research. Under one federated NYU CTSI network, we brought together a leadership team of medical and research directors across the CRCs in Manhattan, Long Island, and Brooklyn to leverage operational and scientific expertise on conducting clinical trials within a culture of safety. Using established CRCs as a model, we replicated standard operating procedures, staffing models, safety monitoring and reporting systems, and training plans. This approach to leadership aligns with the organization’s mission to function as a High Reliability Organization, eliminating preventable harm and streamlining processes to maximize efficiency [19]. In addition to hiring staff from Brooklyn, we provide all CRC nurses and clinical coordinators with both didactic and experiential training in foundational research knowledge. The CTSI also offers training on good clinical practice and responsible conduct of research, as well as best-practice guidance for conducting research at NYULH.

**Figure 2.**
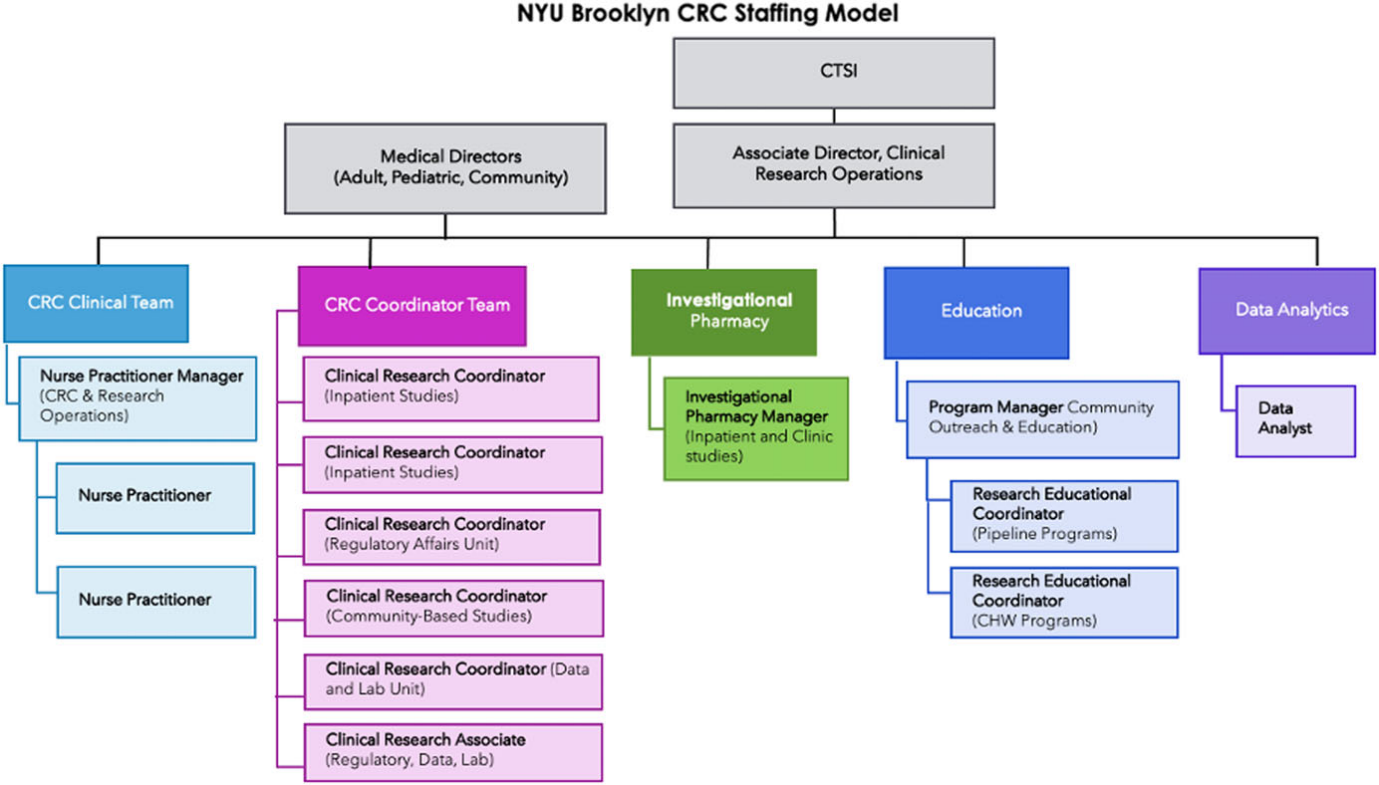
CTSI brooklyn CRC staffing model. Brooklyn CRC organizational staffing chart based on strategic research initiatives and study requirements for inpatient, outpatient, and community-based settings.

Within this framework, we developed a staffing model to support both the current and prospective volume of clinical trials conducted at the CRC, including prioritizing cultural competency, multi-language expertise, and experience working with underserved populations in our hiring strategy (Figure 2). Over half of the CRC staff are bilingual, speaking at least one of the primary languages spoken in the surrounding community, and several are certified as interpreters. Each staff member has prior experience with medically underserved communities or individuals from backgrounds that are underrepresented in medicine. To ensure that staff are equipped with the necessary skills to work with participants from underserved populations, they are required to complete trainings on cultural and language competencies, proper collection of sexual orientation and gender identity data, and empathy awareness workshops [20]. Job opportunities for new roles within the CRC are shared with local organizations to enhance hiring of staff who live within the communities they serve.

To address the FDA draft guidance on DCTs, the Brooklyn CRC offers not only a readily accessible brick-and-mortar site, but we have also partnered with a variety of community-based clinics throughout Brooklyn to enhance the settings in which recruitment and research visits can occur. Nurse practitioners (NPs) can serve as key study personnel to conduct trial activity, thus expanding research capabilities outside of direct faculty oversight [21]. Clinical coordinators are able to travel between the CRC and a network of FHCs; conduct telephone or telemedicine visits whenever possible; and partner with CHWs to broaden enrollment to participants who might otherwise be unfamiliar with clinical trials. Notably, the proximity of the CRC to the hospital contributes to accessibility by enabling participant recruitment in both the inpatient and outpatient settings. Further, this partnership allows adherence to the clinical research as a care option model by ensuring that research encourages and improves standard of care and serves as a treatment option for our hospitalized and clinic patients.

### Evaluation and continuous quality improvement plan

The evaluation and continuous quality improvement plan framework is comprised of three key areas: 1) Implementation (capacity, quality, alignment); 2) linkage (clinical and community research priorities, accessibility and reach, bidirectional exchanges and collaborations with community partners, safety, and monitoring); and 3) impact and sustainability (engagement and outcomes, resources allocation, and funding opportunities). A mixed-methods approach of quantitative and qualitative data collection allowed the team to create and monitor this measure set. We established bi-directional exchanges with our CAB that have led to rapid improvements in our CRC programming and research conducted at our CRC. Ultimately this approach will help create significant and sustainable improvements in access to research, community health outcomes, and the sustainability of our CRC. All analyses, including descriptive statistics, chi-square, T-tests, and linear regressions were performed using R Statistical Software (v4.4.0). Data for studies and patients were pulled from our internal clinical trial management system as well as the electronic health record used for scheduling and documenting research visits.

## Results

Within two years, we established a dedicated CRC and expanded research opportunities to the Brooklyn community. Our CRC is 12,600 square feet, located in a former nursing home, connected to the main hospital by a pedestrian bridge. There are four multipurpose outpatient adult exam rooms, two pediatric exam rooms, a phlebotomy and vitals room, a negative pressure room, an infusion suite, a specimen storage and processing room, and an extended stay room for inpatient studies. Our CRC partners with numerous FHCs and community serving organizations, allowing us to be accessible to the community. Our team includes three medical directors with expertise in adult and pediatric research, an associate director of research, a research NP manager, an investigational pharmacy manager, and four research coordinators. Staffing costs associated with this initiative are $1 million and funded through Beyond Bridges and CTSI, and offset by clinical trial budgets. We have plans to add an additional research NP, a community-based research coordinator, and research education coordinators (Figure 2).

**Figure 3.**
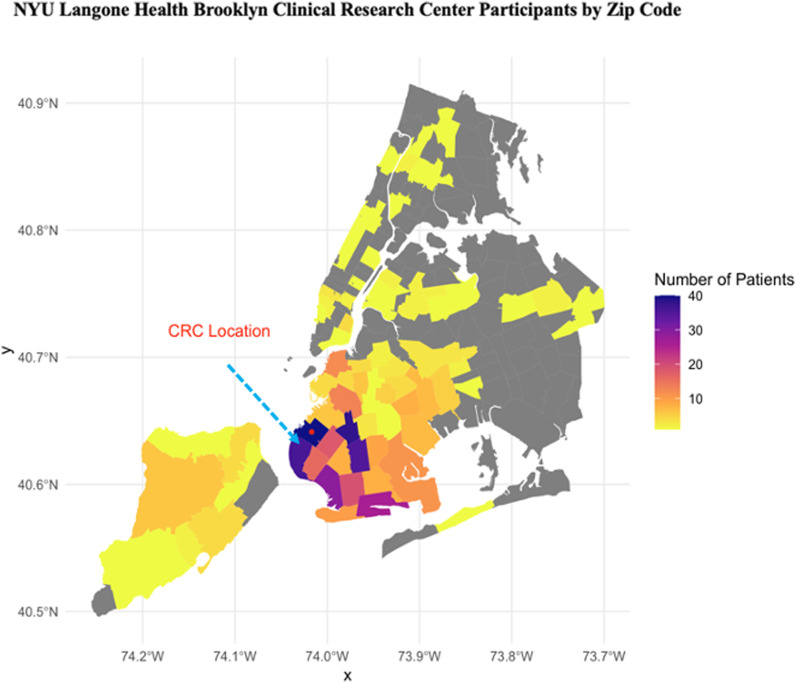
Brooklyn CRC patients by zip code. Number of participants enrolled on to research studies at the brooklyn CRC by zip code.

Within the first two years, the NYULHB CRC has opened 21 studies across 11 different departments, enrolled 509 participants, and served 18 NYULH principal investigators, including 33% early-career and 67% senior faculty (Table 1). In total, 5 studies (24%) were industry funded, 11 studies (52%) were government funded, and 5 studies (24%) were funded through the department, institution, or foundations. Sixty percent of our participants identified as non-White, 20% as Hispanic or Latino, and 28% spoke a language other than English, including Arabic, Bangla, Cantonese, English, Fuzhounese, Mandarin, Polish, Spanish, Urdu, Vietnamese, Italian, and Russian (Table 1). Our participants live in 110 zip codes, 76% are designated HPSA, 66% reside in the immediate CRC neighborhood and surrounding neighborhoods under 5 miles, and 78% are within the borough of Brooklyn (Figure 3). Fifty-four percent of our participants live within 10 miles of our CRC (66% of our participants live within 5 miles or less, 24% live within 5–10 miles), 3% live within 10–15 miles, 3% live within 15–20 miles, and 5% live greater than 20 miles away (Table 1). There was a statistically significant increase from 2022 to 2023 from 13.9% to 25.2% of individuals not residing in Brooklyn attending our CRC, possibly the result of improved community engagement and decentralized recruitment methods, such as virtual consents and follow-up visits conducted remotely.

**Table 1.** Summary statistics for participants enrolled by the brooklyn clinical research center

	Demographics	Overall	2022	2023	p-value
N		509	180	329	
Participant Age (%)	Age	55.50 (17.42)	57.70 (18.66)	54.29 (16.61)	0.035
**Sex (%)**	F	248 (48.8)	101 (56.1)	159 (48.5)	0.120
	M	261 (51.2)	79 (43.9)	169 (51.5)	
**Race (%)**	Asian	150 (29.5)	22 (12.2)	128 (38.9)	<0.001
	Black or African American	82 (16.1)	28 (15.6)	54 (16.4)	
	Native Hawaiian or Other Pacific Islander	3 (0.6)	1 (0.6)	2 (0.6)	
	White	202 (39.7)	89 (49.4)	113 (34.3)	
	More than one race	3 (0.6)	2 (1.1)	1 (0.3)	
	Other	68 (13.4)	38 (21.1)	30 (9.1)	
**Ethnicity (%)**	Hispanic	104 (20.4)	48 (26.7)	56 (17.0)	0.014
	Non-Hispanic	405 (79.6)	132 (73.3)	273 (83.0)	
**Brooklyn Location (%)**	**Living within Brooklyn**	**401 (78.8)**	**155 (86.1)**	**246 (74.8)**	**0.004**
	Living outside of Brooklyn	108 (21.2)	25 (13.9)	83 (25.2)	
**Language (%)**	Arabic	3 (0.6)	1 (0.6)	2 (0.6)	<0.001
	Bengali	28 (5.5)	3 (1.7)	25 (7.6)	
	Cantonese	11 (2.2)	9 (5.0)	2 (0.6)	
	English	369 (72.5)	145 (80.6)	224 (68)	
	Fuzhounese	2 (0.4)	1 (0.6)	1 (0.3)	
	Italian	1 (0.2)	0 (0.0)	1 (0.3)	
	Mandarin	2 (0.4)	1 (0.6)	1 (0.3)	
	Polish	1 (0.2)	1 (0.6)	0 (0.0)	
	Russian	1 (0.2)	0 (0.0)	1 (0.3)	
	Spanish	35 (6.9)	18 (10.0)	17 (5.2)	
	Urdu	55 (10.8)	0 (0.0)	55 (16.7)	
	Vietnamese	1 (0.2)	1 (0.6)	0 (0.0)	
**Health Professional Shortage Area Zip Codes (%)**	Yes (%)	386 (75.8)	136 (75.6)	250 (76.0)	0.999
	No (%)	123 (24.2)	44 (24.4)	79 (24.0)	
**Distance from CRC**	0-5 mile	332 (65.5)	133 (73.9)	199 (60.9)	0.012
	5-10 mile	120 (23.7)	38 (21.1)	82 (25.1)	
	10-15 mile	15 (3.0)	3 (1.7)	12 (3.7)	
	15-20 mile	17 (3.4)	3 (1.7)	14 (4.3)	
	20+ mile	23 (4.5)	3 (1.7)	20 (6.1)	

Table of descriptive statistics of patients enrolled onto clinical trials at the Brooklyn clinical research center.

## Discussion

The establishment of the NYULHB CRC represents a stride toward addressing health disparities by increasing research accessibility in a HPSA neighborhood in Brooklyn, New York. The implementation and scalability of our initiatives required inter- and intradepartmental collaboration, sharing of resources, as well as buy-in from a variety of multi-sector partners across the NYULH health system and Sunset Park community. The process began with a planning phase to assess the healthcare needs of the community and align clinical and community priorities with research, followed by the development of a strategic plan that outlines steps to achieving improved outcomes. An internal survey and listening sessions with physicians in Brooklyn identified the need for physical infrastructure, including development of research and community space, the establishment of clinical and research priorities, as well as education and training initiatives. FHC feedback identified the need for both financial and personnel support for busy front-line clinicians to conduct research on FHC disease priorities directly within FHC clinics. Future work will further assess the feasibility of integrating CRC operations within FHC settings.

Following the assessment, the plan included the establishment of a robust infrastructure, creation of an expert research leadership team, recruitment of a dedicated, experienced, culturally competent, and linguistically appropriate team of research professionals, and the creation of partnerships with local stakeholders including the FHC and community partners. The implementation phase involved executing the strategic plan with continuous monitoring and evaluation to ensure the unit’s effectiveness and sustainability. This included gathering feedback from front-line providers on expanding SDOH support through social workers and embedding a community coordinator to help facilitate and navigate research participants from the community and FHC clinics into research trials. This endeavor is not without its challenges, including limited financial resources, cultural and language barriers, and the need for increased direct community engagement. However, with careful planning, collaboration, and a commitment to serving the community, the development and implementation of an academic CRC in an underserved area can be accomplished with the goals of improving healthcare outcomes and advancing medical research in densely populated settings. This approach may not be reproducible in suburban or sparsely populated rural settings.

The successful implementation of our CRC can serve as a model for establishing similar centers nationwide, particularly in underserved and resource-poor, HPSA communities. By leveraging a community-centered approach, creating robust research infrastructure, and assembling a dedicated, culturally competent team, other regions can replicate this model to address local health disparities and access to research. The deployment of DCTs, especially in resource-poor and rural communities, can further enhance accessibility and allow for a broader reach. Facilitators to implementation include 1) strong community partnerships and engagement; 2) sustainable funding support; 3) robust infrastructure and collaboration with academic medical centers; 4) recruitment of a culturally competent, multilingual, and expert clinical research staff; 5) implementation and expansion of DCT frameworks in all research projects; and 6) continuous evaluation and quality improvement. National implementation would require collaboration with local academic healthcare systems, community organizations, foundations, and federal agencies to ensure adequate funding, resource allocation, and adherence to best practices in clinical research.

The successful initiation of numerous trials across diverse medical disciplines underscores the center’s commitment to providing cutting-edge research opportunities to underserved populations. Based on our priorities and strategic plan, we have hired staff that speak the languages of the community, are culturally competent with the communities served, and understand the intricacies of our borough. Furthermore, navigating cultural barriers and fostering community engagement have emerged as critical considerations, emphasizing the necessity of building trust and fostering collaborative relationships within the healthcare system and community, including NYULHB, the FHC clinics, and the community. The high recruitment and demographic diversity serve as a testament to the center’s efficacy in engaging and involving our healthcare enterprise and external community. Moreover, it illustrates a further need of creating physical access to clinical research in underserved and underrepresented communities and decentralizing research closer to where participants live. A large, national study evaluating race and ethnicity data among 20,692 trials found that only 43% of trials reported race and ethnicity over a 10-year period and, of those that did report it, close to 80% enrolled White populations, followed by 10% African American, 6% Hispanic and only 1% Asian [22]. Our center overwhelmingly enrolled non-White populations and endeavored to collect race, ethnicity, language, gender, and location data on every enrollee. Future studies, including comparative research with other CRCs, would help validate this community-centered approach.

While further collaborations with multiple stakeholders will be required to expand research beyond the CRC and into the community, establishing an informal practice-based research network comprising of NYULH family group practices, FHC clinics, and community-based organizations, the community-centered CRC brings the NYU CTSI one step closer to improving health equity for the communities that are most in need. Additionally, future surveys and interviews with staff, community organizations, faculty researchers, and front-line providers will enable us to evaluate the need for further expansion and programmatic efforts to enhance clinical research, taking into account community and clinical priorities. Our expected growth of 24 additional studies will explore innovative therapies in cardiovascular disease, stroke, gestational diabetes, long COVID, diabetes, autoimmune disorders, dementia, pneumonia, obesity, and cancer genetic screening, all which are in line with our Research Priority Model. Plans for expansion also include the hiring of additional clinical and community-based research staff to support our growing research portfolio, with a focus on bridging our community-clinic linkage model.
